# Analysis of fluorescent reporters indicates heterogeneity in glucose uptake and utilization in clonal bacterial populations

**DOI:** 10.1186/1471-2180-13-258

**Published:** 2013-11-15

**Authors:** Nela Nikolic, Thomas Barner, Martin Ackermann

**Affiliations:** 1 Institute of Biogeochemistry and Pollutant Dynamics, Department of Environmental Systems Science, ETH Zurich, Zurich, Switzerland; 2 Department of Environmental Microbiology, Eawag, Duebendorf, Switzerland; 3 Current address: Max F. Perutz Laboratories, Department of Microbiology, Immunobiology and Genetics, University of Vienna, Vienna, Austria

**Keywords:** Phenotypic variation, Glucose, Acetate, Uptake, Metabolism, Cross-feeding, Fluorescent reporters, Flow cytometry

## Abstract

**Background:**

In this study, we aimed at investigating heterogeneity in the expression of metabolic genes in clonal populations of *Escherichia coli* growing on glucose as the sole carbon source. Different metabolic phenotypes can arise in these clonal populations through variation in the expression of glucose transporters and metabolic enzymes. First, we focused on the glucose transporters PtsG and MglBAC to analyze the diversity of glucose uptake strategies. Second, we analyzed phenotypic variation in the expression of genes involved in gluconeogenesis and acetate scavenging (as acetate is formed and excreted during bacterial growth on glucose), which can reveal, for instance, phenotypic subpopulations that cross-feed through the exchange of acetate. In these experiments, *E. coli* MG1655 strains containing different transcriptional GFP reporters were grown in chemostats and reporter expression was measured with flow cytometry.

**Results:**

Our results suggest heterogeneous expression of metabolic genes in bacterial clonal populations grown in glucose environments. The two glucose transport systems exhibited different level of heterogeneity. The majority of the bacterial cells expressed the reporters for both glucose transporters MglBAC and PtsG and a small fraction of cells only expressed the reporter for Mgl. At a low dilution rate, signals from transcriptional reporters for acetyl-CoA synthetase Acs and phosphoenolpyruvate carboxykinase Pck indicated that almost all cells expressed the genes that are part of acetate utilization and the gluconeogenesis pathway, respectively. Possible co-existence of two phenotypic subpopulations differing in *acs* expression occurred at the threshold of the switch to overflow metabolism. The overflow metabolism results in the production of acetate and has been previously reported to occur at intermediate dilution rates in chemostats with high concentration of glucose in the feed.

**Conclusions:**

Analysis of the heterogeneous expression of reporters for genes involved in glucose and acetate metabolism raises new question whether different metabolic phenotypes are expressed in clonal populations growing in continuous cultures fed on glucose as the initially sole carbon source.

## Background

This study focuses on the analysis of reporters for the expression of metabolic genes as a first step towards the analysis of phenotypic variation in metabolism in clonal populations of *Escherichia coli*. Our aim was to explore whether different systems that transport glucose exhibit different level of heterogeneity. We were also interested in whether certain conditions promote heterogeneity further downstream, in metabolic reactions. We therefore investigated if bacterial populations growing on solely glucose form phenotypically distinct subpopulations that express different parts of metabolic pathways. The rationale for these analyses was that, even under constant and homogeneous conditions, single cells can show marked differences in phenotypic traits [1,2], including the expression of different transporters and metabolic enzymes. Such phenotypic variation can arise through a number of cellular processes; one well-studied phenomenon is ‘stochastic gene expression’ [3], i.e. the fact that many cellular processes are inherently variable, and that this can lead to substantial phenotypic variation that is produced independently of genetic or environmental differences [1,4,5].

Generally, variation in gene expression can have functional consequences and provide adaptive benefits. In situations in which the environment changes rapidly, genotypes that produce higher levels of phenotypic variation among individuals can have a higher probability to thrive [6-8]. In this study, we focus on cases in which variation in gene expression might potentially provide a different benefit. In some scenarios, it might be advantageous for cells to specialize in their metabolic function [9], for example due to inefficiencies or trade-offs [10] that arise from performing different metabolic functions within the same cell. In such cases, we might expect that individual cells within a population will either perform one function or the other, but not both. To test for instances in which we find metabolic specialization, we analyzed gene expression as a proxy for how glucose and acetate metabolism differs between single cells in clonal populations grown in glucose environments.

Previous studies have established that *E. coli* can employ different transport systems to take up a given carbon source from the environment. The redundancy in glucose (Glc) uptake has, in particular, been widely studied. *E. coli* can use five different permeases for glucose, which belong to three protein families: MglBAC is an ABC (ATP-binding cassette) transporter; GalP is a MFS (major facilitator superfamily) transporter; and PtsG/Crr, ManXYZ and NagE are parts of PTS (phosphotransferase system) [11-13]. Population-based studies have shown that the expression of a specific glucose transporter highly depends on the bacterial growth rate and the concentration of glucose in the environment [11,12]. PtsG/Crr is the only glucose-specific PTS permease (Glc-PTS) and transcription of *ptsG* is induced solely by glucose [14]. MglBAC is an uptake system that is induced by glucose and galactose, whereas GalP exhibits a wider range of specificity as it can transport different carbon sources. MglBAC and PtsG/Crr are the uptake systems that engage in most of the glucose transport in *E. coli* in different glucose environments [11,12,14-16]. The Mgl system has the leading role in glucose uptake in carbon-limited chemostat cultures. *mgl* expression increases rapidly at the onset to glucose depletion, particularly in glucose-limited chemostat cultures [15,16]. MglBAC additionally allows bacteria to utilize glucose in micromolar concentrations. It is the most highly expressed transporter under glucose limitation [11] due to its high affinity for glucose [12], but PTS also transports glucose with similar micromolar affinity [12,17,18]. Regarding dependence of activity of glucose transporters on bacterial growth rate, at intermediate growth rates Mgl has the leading role in glucose uptake, although PtsG is active as well [15]. Regulation of expression and activity of transporters PtsG/Crr and MglBAC is substantially different. Different groups of sigma factors, activators and repressors are responsible for regulation of their transcription, including a small RNA that additionally controls degradation of the *ptsG* transcript [12,14,19]. Furthermore, PtsG/Crr takes up and concomitantly phosphorylates glucose in an ATP-independent fashion, whereas glucose transported via ATP-dependent uptake system MglBAC is subsequently phosphorylated by a different enzyme [12].

Glucose is metabolized via central metabolism, which is the source of energy and biomass building blocks. First, the glycolytic enzymes break down glucose to pyruvate, which is then further metabolized to acetyl-CoA that can enter the citric acid cycle [20]. If glucose is present in the environment as a sole carbon source, cells growing at a high rate of glucose consumption perform a fast metabolism known as overflow metabolism [21]. The cells rapidly degrade glucose to acetyl-CoA and further to acetate, and ultimately excrete acetate [22]. Two different pathways can catalyze the excretion of acetate: Pta-AckA (phosphate acetyltransferase - acetate kinase) during the exponential phase or PoxB (pyruvate oxidase) in the stationary phase [23,24]. Furthermore, *E. coli* also has the ability to grow on acetate as a sole carbon source [21]. Acetate can freely penetrate the cell membrane [21] but it also has its dedicated uptake system ActP (acetate permease) that is co-transcribed with *acs* encoding for acetyl-CoA synthetase [25]. Bacteria utilize acetate by using the low affinity Pta-AckA pathway when acetate is present in high concentrations in the millimolar range. Acetyl-CoA synthetase Acs takes over acetate uptake at low concentrations of acetate in the micromolar range [21,26]. However, the growth rate when growing solely on acetate is low: for example, the maximal growth rate on acetate is almost five times lower than on a concentration of glucose with the equivalent number of carbon atoms [27]. In batch cultures with glucose as the sole provided carbon source, *E. coli* populations start to grow on the excreted acetate when glucose is depleted [21].

As mentioned above, acetate appears as an intermediate in reactions of glucose metabolism, and it can as well serve as a carbon source. This raises the question whether clonal bacterial populations growing in constant glucose-feed conditions form two physiologically different groups: one group that excretes acetate and has *acs* down-regulated and a second group that expresses *acs* and utilizes acetate. Such a situation would correspond to phenotypic cross-feeding. The term cross-feeding describes a metabolic interaction where the complete degradation of a substrate is partitioned between two types. One type utilizes a nutrient from the environment (e.g. glucose) and excretes the metabolized product (e.g. acetate) that is afterwards used as the primary nutrient source for the second type. Previous studies have only focused on cross-feeding between different genotypes within bacterial populations, which can spontaneously evolve in experimental microbial populations growing on glucose as the sole carbon source [28,29]. In this study, we hypothesized that cross-feeding could also arise within an isogenic bacterial population, based on the emergence of phenotypic subpopulations with different expression of metabolic genes. Acetate cross-feeding subpopulations could potentially occur in glucose-fed clonal populations and scavenge acetate that is excreted by other cells.

## Results and discussion

### Different levels of phenotypic variation between different glucose transporters

Our focus was on quantifying heterogeneity in the expression of genes involved in the uptake and utilization of glucose and its metabolic intermediate acetate. We used a plasmid-based reporter system [30] in which fluorescence from promoter-*gfp* fusion constructs serves as an indirect measurement of transcription. In our recent work [31], we showed that signals from such plasmid-based fluorescent reporters were significantly correlated with directly measured levels of mRNA as well as with measurements of translational reporters [32], although the latter association was weaker. Analyses of the fluorescence of promoter-*gfp* reporters therefore provide partial (but not complete) information about the actual expression of a gene. We also established [31] that using this plasmid-based reporter system [30] gives comparable results of mean and variation of expression to reporter systems integrated into the chromosome.

**Figure 1:**
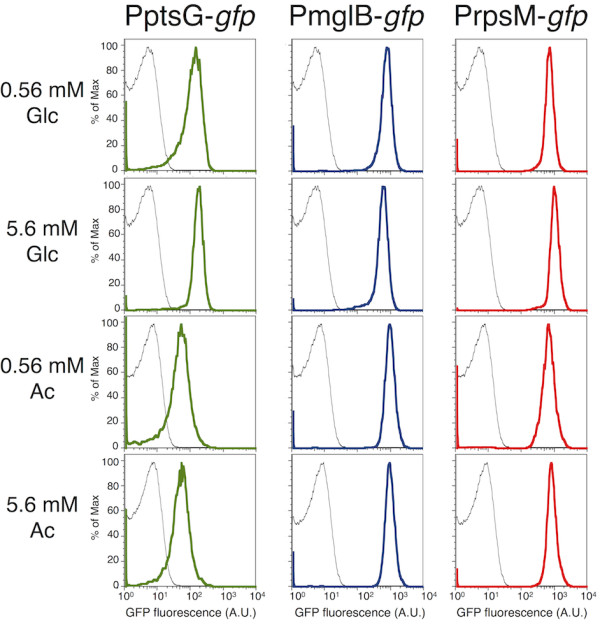
**Expression of *ptsG*, *mglB *and *rpsM *reporters at D = 0.15 h**^**-1**^**.** Fluorescence measurements represent expression of PptsG*-gfp* (green), PmglB*-gfp* (blue), PrpsM*-gfp* (red) and negative control (black). Bacteria were grown in minimal media supplemented with different concentrations of D-glucose (Glc) or sodium acetate (Ac). The variation in expression of the *ptsG* reporter is higher than the variation in expression of the *mglB* reporter.

We first investigated variation in the expression of reporters for the transporters PtsG and MglBAC, which are the most prominent glucose uptake systems in *E. coli*[12,15,16]. The aim was to test whether these glucose transporters exhibit different levels of heterogeneity in gene expression. The expression of *ptsG* and *mglB* reporters was measured in media supplemented solely with glucose (see Methods; the results are shown in Table 1, Table 2 and Additional file 1: File S1). The mean expression of PmglB-*gfp* was higher than PptsG-*gfp* in all tested glucose growth conditions (Table 1), which is consistent with previous reports that MglBAC is the most highly expressed glucose transporter at intermediate growth rates [15]. Next, we aimed to assess whether different glucose transport strategies exhibit different levels of variation in gene expression. We used two different approaches to do so. First, we computed the coefficient of variation (CV, the ratio between the standard deviation and the mean) for each measurement of GFP fluorescence. As control, we used the reporter for *rpsM*, which encodes the ribosomal protein S13, previously shown to exhibit a low degree of variation in the expression between clonal cells [31]. The *ptsG* reporter showed higher CVs than the *mglB* reporter in all glucose-feed environments (Table 2, Additional file 1: File S1), and also higher CVs than the PrpsM-*gfp* control (Figure 1, Table 2). However, CVs alone are not a reliable indicator for the level of heterogeneity in gene expression, since it has been previously demonstrated that CVs are dependent on the mean expression level [31]. This relationship also manifests in our dataset in all tested growth conditions (presented in the next section of Results and Discussion).

**Table 1:** Values for mean log expression of measured reporter strains

		**Mean log expression**		
**Experimental conditions**	***ptsG***	***mglB***	***rpsM***	***acs***
Chemostat, D = 0.15 h^-1^; 0.56 mM Glc	1.94 ± 0.02	2.78 ± 0.01	2.84 ± 0.03	2.18 ± 0.02
Batch; 0.56 mM Glc	2.05 ± 0.02	2.19 ± 0.01	3.14 ± 0.01	1.90 ± 0.02
Chemostat, D = 0.3 h^-1^; 0.56 mM Glc	2.11 ± 0.06	2.75 ± 0.02	2.78 ± 0.09	2.12 ± 0.01
Chemostat, D = 0.15 h^-1^; 5.6 mM Glc	2.18 ± 0.03	2.75 ± 0.03	2.97 ± 0.01	1.93 ± 0.02
Batch; 5.6 mM Glc	1.94 ± 0.02	2.25 ± 0.04	3.25 ± 0.00	1.50 ± 0.06
Chemostat, D = 0.15 h^-1^; 0.56 mM Ac	1.36 ± 0.04	2.83 ± 0.05	2.65 ± 0.02	2.24 ± 0.00
Batch; 0.56 mM Ac	1.44 ± 0.03	2.80 ± 0.02	2.81 ± 0.03	1.97 ± 0.16
Chemostat, D = 0.15 h^-1^; 5.6 mM Ac	1.57 ± 0.02	2.87 ± 0.02	2.81 ± 0.03	2.18 ± 0.02
Batch; 5.6 mM Ac	1.19 ± 0.00	2.85 ± 0.02	2.82 ± 0.03	1.91 ± 0.01
Chemostat, D = 0.15 h^-1^; 2.8 mM Glc, 2.8 mM Ac	2.02 ± 0.02	2.78 ± 0.08	2.78 ± 0.01	2.04 ± 0.00
Batch; 2.8 mM Glc, 2.8 mM Ac	1.96 ± 0.01	2.23 ± 0.02	3.20 ± 0.04	1.66 ± 0.01
Chemostat, D = 0.15 h^-1^; 0.28 mM Glc, 0.28 mM Ac	1.71 ± 0.04	2.81 ± 0.02	2.74 ± 0.02	2.06 ± 0.02
Batch; 0.28 mM Glc, 0.28 mM Ac	1.98 ± 0.002	2.37 ± 0.02	3.11 ± 0.02	1.85 ± 0.01

The values are represented as mean of the replicates ± standard error of the mean.

**Table 2:** Values for CV of log expression of measured reporter strains

		**CV of log expression**		
**Experimental conditions**	***ptsG***	***mglB***	***rpsM***	***acs***
Chemostat, D = 0.15 h^-1^; 0.56 mM Glc	0.21 ± 0.02	0.17 ± 0.01	0.13 ± 0.02	0.14 ± 0.02
Batch; 0.56 mM Glc	0.12 ± 0.01	0.08 ± 0.00	0.06 ± 0.00	0.14 ± 0.00
Chemostat, D = 0.3 h^-1^; 0.56 mM Glc	0.25 ± 0.01	0.15 ± 0.01	0.19 ± 0.07	0.11 ± 0.01
Chemostat, D = 0.15 h^-1^; 5.6 mM Glc	0.15 ± 0.01	0.11 ± 0.01	0.08 ± 0.01	0.15 ± 0.01
Batch; 5.6 mM Glc	0.10 ± 0.01	0.10 ± 0.01	0.07 ± 0.01	0.24 ± 0.02
Chemostat, D = 0.15 h^-1^; 0.56 mM Ac	0.46 ± 0.03	0.22 ± 0.03	0.25 ± 0.01	0.22 ± 0.00
Batch; 0.56 mM Ac	0.47 ± 0.02	0.22 ± 0.01	0.20 ± 0.03	0.38 ± 0.10
Chemostat, D = 0.15 h^-1^; 5.6 mM Ac	0.28 ± 0.01	0.17 ± 0.01	0.21 ± 0.02	0.19 ± 0.02
Batch; 5.6 mM Ac	0.64 ± 0.00	0.20 ± 0.01	0.23 ± 0.00	0.41 ± 0.01
Chemostat, D = 0.15 h^-1^; 2.8 mM Glc, 2.8 mM Ac	0.19 ± 0.01	0.18 ± 0.03	0.18 ± 0.03	0.19 ± 0.02
Batch; 2.8 mM Glc, 2.8 mM Ac	0.09 ± 0.00	0.07 ± 0.00	0.05 ± 0.01	0.19 ± 0.00
Chemostat, D = 0.15 h^-1^; 0.28 mM Glc, 0.28 mM Ac	0.23 ± 0.01	0.15 ± 0.03	0.18 ± 0.04	0.22 ± 0.01
Batch; 0.28 mM Glc, 0.28 mM Ac	0.11 ± 0.02	0.08 ± 0.00	0.08 ± 0.01	0.15 ± 0.00

The values are represented as mean of the replicates ± standard error of the mean.

We thus used a second measure for variation in gene expression: the fraction of cells in a clonal population that expressed the transcriptional reporter above background levels. We subtracted the background fluorescence (log_10_ value of 1.3; see Methods) from the measurements of expression of PptsG-*gfp* and PmglB-*gfp*, for all growth conditions that we tested. Expression of PmglB-*gfp* was above background in 90.1-99.8% of the cells within a population (one measurement for each environmental condition presented in Table 3; Additional file 1: File S1), depending on the growth conditions. This implies that the vast majority of cells transcribe *mglBAC* regardless of the carbon sources present in the media or the growth rate. Considering only cultures grown on glucose, 96.8-99.8% of the population expressed the *mglB* reporter above background. In the same conditions, the fraction of cells that did not express PptsG-*gfp* was in two cases above 5%. For instance, 8.6% of the cells in the population that was grown in the chemostats cultures [33] at D = 0.15 h^-1^ with 0.56 mM Glc did not express PptsG-*gfp*. It is conceivable that a subfraction of the cells that do not express PptsG-*gfp* is metabolically inactive. To test this, we compared the fraction of cells that does not express PptsG-*gfp* with the fraction of cells that does not express the ribosomal reporter PrpsM-*gfp*, measured under the same conditions. The ribosomal reporter indicated that only around 0.5% of the population did not transcribe the ribosomal protein (Table 3), i.e. those were probably dead or not actively dividing cells. This indirectly implies that most of the cells that did not express PptsG-*gfp* may be metabolically active and should thus engage in another glucose uptake strategy.

**Table 3:** Percentage of cells within a population that expressed the reporters above the background level

**Experimental conditions**	***rpsM***	***ptsG***	***mglB***
Chemostat, D = 0.15 h^-1^; 0.56 mM Glc	99.5	91.4	96.8
Batch; 0.56 mM Glc	99.7	99.2	99.7
Chemostat, D = 0.3 h^-1^; 0.56 mM Glc	99.7	82.2	97.7
Chemostat, D = 0.15 h^-1^; 5.6 mM Glc	99.6	96.9	98.7
Batch; 5.6 mM Glc	99.7	98.9	99.8
Chemostat, D = 0.15 h^-1^; 0.56 mM Ac	93.9	71.4	90.1
Batch; 0.56 mM Ac	92.1	76.0	94.1
Chemostat, D = 0.15 h^-1^; 5.6 mM Ac	98.4	84.9	96.3
Batch; 5.6 mM Ac	94.6	83.2	96.6
Bhemostat, D = 0.15 h^-1^; 2.8 mM Glc, 2.8 mM Ac	99.0	97.2	93.5
Batch; 2.8 mM Glc, 2.8 mM Ac	99.8	99.5	99.8
Chemostat, D = 0.15 h^-1^; 0.28 mM Glc, 0.28 mM Ac	99.5	91.9	92.8
Batch; 0.28 mM Glc, 0.28 mM Ac	99.1	99.3	99.6

Overall, these results suggest that the promoter for *mglBAC* is expressed above background in a higher fraction of the population than the promoter for *ptsG*, and differences in *ptsG* expression between genetically identical cells could be an indication of glucose uptake heterogeneity within clonal populations.

**Figure 2:**
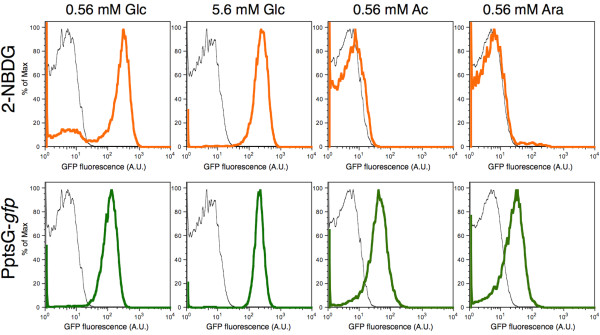
**Comparison of Glc-PTS activity and PptsG-*gfp *expression in different chemostat conditions.** The distributions show Glc-PTS (PtsG/Crr) activity (orange) based on uptake of a fluorescent glucose analog, expression of PptsG*-gfp* (green) and negative control (wild-type MG1655, black). Bacteria were grown in minimal media supplemented with D-glucose (Glc), sodium acetate (Ac) or L-arabinose (Ara), at dilution rate D = 0.15 h^-1^. Some cells expressed the *ptsG* reporter in conditions when no glucose was taken up via Glc-PTS. Also, low concentration of glucose in the medium feed (first column) led to the existence of a small subpopulation that does not engage in the glucose uptake via Glc-PTS.

Next, we used direct measurements of uptake to analyze the activity of the glucose-PTS transporter and to compare the transporter activity with the expression of PptsG-*gfp*. 2-NBDG, 2-[N-(7-nitrobenz-2-oxa-1,3-diazol-4-yl)amino]-2-deoxy-D-glucose, is a fluorescent D-glucose analog, and has been used to study the dynamics of glucose uptake via the phosphotransferase system (PTS) in single cells of *E. coli*[18,34]. Since 2-NBDG is exclusively taken up via Glc-PTS, cells will fluoresce only if their PTS system is active and the glucose analog is transported inside the cell. As this assay uses a glucose analog that cannot be metabolized, the results can be interpreted only in the context of the activity of the transport system and not as a general measure of metabolic activity of a cell. Our data indicate that not all cells use the PTS system to take up glucose from the media (Figure 2, medium supplemented with 0.56 mM Glc). How do the rest of the cells take up glucose – do they maybe employ alternative carbon sources? There are two possibilities. First, cells might use Mgl or another glucose transporters. Second, it is possible that the cells use excreted acetate as (an additional) carbon source. We also found that even if the PptsG-*gfp* reporter strain fluoresces, it does not necessarily mean that PTS is actively transporting glucose (Figure 2). This became evident in control experiments where we grew cells in medium containing acetate or arabinose as the sole carbon source. Around 80% of the gated population growing in acetate (around 60% growing in arabinose) expressed the *ptsG* reporter above the background level, without any glucose present to induce the expression or to be transported (Additional file 1: File S1). Furthermore, in these conditions the PptsG-*gfp* reporter showed a high degree of variation in expression (Figure 2).

Transcriptional reporters for glucose transporters can only provide limited insights into the actual metabolic state of cells. Several recent papers have discussed discrepancies between transcriptional reporters and metabolic fluxes in specific parts of metabolic pathways [35,36]. As a consequence, we need to be cautious when using data from transcriptional reporters to make inferences about the actual physiology of cells. Additional experiments could provide complementary insights, for instance the analysis of sugar transporter synthesis or activity, together with analysis of sugar assimilation at the single-cell level [37].

### Variation in the expression of glucose transporters across environments

**Figure 3:**
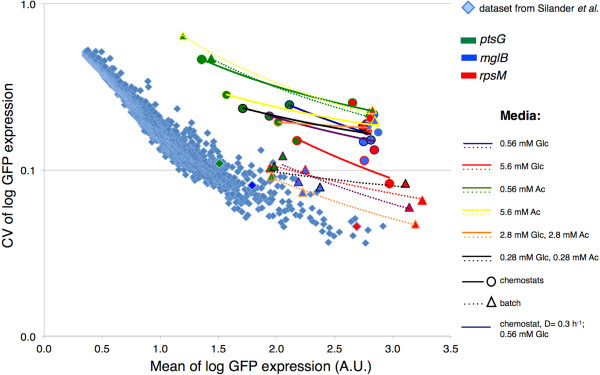
**Phenotypic variation in gene expression in 13 different environments.** The coefficient of variation (CV) of log expression of PptsG-*gfp*, PmglB-*gfp* and PrpsM-*gfp* was plotted against the mean log expression. Expression of the reporters in different environments was compared to data for 1522 *E.coli* promoters [31] (light blue diamonds) that were measured in the early exponential phase in batch cultures containing arabinose as a sole carbon source. Circles represent measurements in chemostat environments and triangles represent measurements in batch cultures. Different color of triangles and circles represents different reporters: *ptsG* (green), *mglB* (blue) and *rpsM* (red). Power regression (i.e. linear regression on log-transformed data) was fitted to each set of three promoters measured in the same environment. Colors of fitted lines mark different carbon sources in the feed; full lines mark chemostat environments and dashed lines mark batch cultures. Each data point is the average over 2–5 independent replicates (except for data from [31]).

We next investigated how the variation in expression of reporters for different glucose transporters changes across different environments. We first compared the results of this study with the results from a genome-wide study of promoter-mediated phenotypic variation [31]. Mean and variation of the expression of *ptsG*, *mglB* and *rpsM* reporters are shown in Figure 3 (plotted are mean values of replicates in different conditions). When power regression lines were fitted across different expression data from the same environment, all lines showed the same trend, namely that the CV of log fluorescence values decreased with mean log GFP expression (Figure 3). Our analysis suggests some general rules: variation in the expression from these three promoters was lowest in batch cultures supplemented with glucose, or glucose plus acetate, and highest in batch or chemostats cultures with acetate as a sole carbon source.

Our results also show that, while mean and variation of reporter expression in a given environment follow a clear and simple pattern (Figure 3), measurements of mean and variation in expression of a particular reporter in different environments deviate substantially from this pattern (Additional file 2: Figure S1). The specifics of these deviations were dependent on the reporter we analyzed: *ptsG* showed a negative association between mean expression and the variation of expression across environments, while *mglB* showed a positive association. We speculate that these differences between *ptsG* and *mglB* could be a consequence of distinctive regulatory features of the glucose transporters [12-15,17,19], different affinity towards transported sugar [12,17], and possibly different growth rate dependencies [38].

### Variation in the expression of genes involved in glucose and acetate utilization

**Figure 4:**
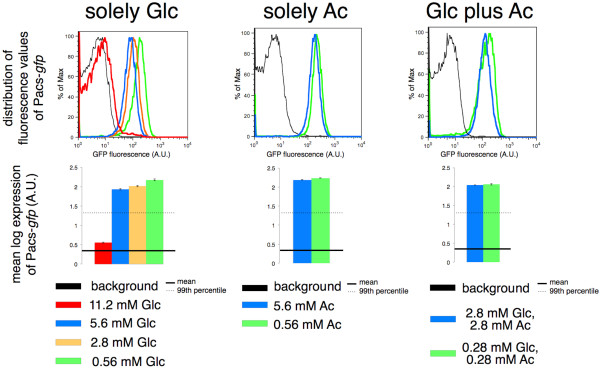
**Expression of the *acs *reporter in different chemostat environments at D = 0.15 h**^**-1**^**.** Fluorescence measurements report the expression of Pacs-*gfp* in chemostat environments supplied with minimal media supplemented with only D-glucose, only sodium acetate or D-glucose plus sodium acetate. Background fluorescence is the fluorescence of the promoterless strain depicted in black. The error bars on the plots for mean log expression of Pacs-*gfp* are standard errors of the mean. The expression of the *acs* reporter was down-regulated to the greatest extent in chemostats with high concentration of glucose (11.2 mM Glc in the feed).

Besides exhibiting heterogeneity in uptake of glucose, cells could show phenotypic variation in the expression of metabolic genes involved in utilization of glucose and acetate. In particular, we were interested in gene expression patterns that could indicate variation between cells in the consumption of acetate; in our system, acetate can come from two different sources – from the same cell or taken up from the environment where it is excreted by other cells. As discussed in the Background, the presence of cells that take up acetate produced by other cells would be indicative of phenotypic cross-feeding in clonal populations. To investigate this, we constructed a Pacs-*gfp* reporter to measure the expression of the gene encoding for acetyl-CoA synthetase Acs. Generally, rapid increase in *acs* transcription occurs when bacterial cultures are inoculated into medium containing solely acetate as a carbon source [26]. The promoter Pacs controls the *acs-yjcH-actP* operon, and hence also controls transcription of the acetate permease ActP [25]. Therefore, differential regulation of *acs* can also indicate altered expression of the acetate transporter and regulation of the uptake of external acetate. However, uptake via ActP is not the only acetate uptake strategy, since acetate can freely diffuse into cells [21]. The expression of *acs* is down-regulated when bacteria excrete acetate [39] and up-regulated when bacteria utilize acetate [40]. Accordingly, we detected increased expression of the *acs* reporter when bacteria were grown only on acetate in comparison to growth on glucose (Figure 4, Additional file 1: File S1). Moreover, the expression of the *acs* reporter was reduced when the concentration of glucose in the chemostat feed was increased (Figure 4). This is consistent with previous reports that have shown that high concentrations of glucose lead to an increase in the intracellular concentration of acetate [39], resulting in down-regulation of the *acs* operon. Furthermore, we observed that variation in Pacs-*gfp* expression was actually lower in glucose-limited chemostats than in acetate-limited continuous cultures (Table 2) or in glucose-acetate continuous cultures (Additional file 3: Text S1, Additional file 4: Figure S2).

Results from previous studies suggest that under the conditions used here – glucose as the only carbon source, and low dilution rates – the reactions of glyoxylate shunt and gluconeogenesis should be active, which would allow utilization of simple carbon sources such as acetate when glucose is not available [20]. According to population-based studies on bacteria grown on glucose, the shunt operates at the dilution rates from 0.05–0.2 h^-1^, allowing metabolism of acetyl-CoA to succinate. The reactions of the citric acid cycle are not engaged, and this prevents carbon loss in the form of CO_2_[33,41]. When acetate is used as a sole carbon source, the expression of the phosphoenolpyruvate (PEP) carboxykinase gene *pck* (a gluconeogenesis enzyme) is up-regulated [40,42], indicating synthesis of glucose from non-carbohydrate precursors such as acetate [20]. *pck* is also up-regulated in chemostats containing glucose as a carbon source that are run at low dilution rates [43].

Our experiments at the single-cell level largely support these previous population-based studies. In the following paragraph, we will discuss in more details the gene expression phenotypes that we observed in clonal populations grown in mini-chemostats at low dilution rate of D = 0.15 h^-1^, and with glucose as the sole carbon source at a feed concentration of 0.56 mM Glc. These are the conditions in which the majority of the cells expressed both glucose transporters *mglB* and *ptsG*, whereas some cells only expressed *mglB* (Figure 1, Table 3). The fraction of cells that did not express the ribosomal reporter was below 1% (Table 3), and these were the cells that presumably did not grow and divide. The residual concentration of glucose in the mini-chemostats after five volume changes (theoretical steady-state concentration [33]) was 1.95 ± 0.13 μM, measured by ion chromatography (our experimental setup did not allow us to accurately measure concentration of acetate).

We found that, under these conditions, almost all cells expressed the *acs* reporter above background level (Figure 4). This may indicate that they either recover cytoplasmic acetate or take up acetate excreted by others. Recovering cytoplasmic acetate can be consequence of homeostasis of acetyl-AMP and acetylphosphate [44]. In chemostats run under such conditions, acetate is usually not detected [43-45], however it might be possible that scarce amounts of acetate are excreted and immediately taken up by an acetate cross-feeding subpopulation. It has been argued that the production of acetate is independent of the growth rate and that the growing bacteria can simultaneously produce and utilize acetate [45,46]. The expression of the *pck* reporter also indicates that most of the cells possibly engaged in the reactions of gluconeogenesis (Additional file 5: Figure S3).

Previous studies provided evidence that transcriptional regulation does indeed have a significant impact on the direction of the metabolic flux through the pyruvate/acetyl-CoA node [36]. Transcriptional control at this branching point allows flux to proceed via overflow metabolism, citric acid cycle and/or PEP-glyoxylate cycle [35]. Results presented in another paper indicate that alterations of fluxes through the glyoxylate shunt and the citric acid cycle were associated with changes in the expression of these genes [47]. Therefore, transcriptional reporters for acetate metabolism (the *acs* reporter) and PEP-glyoxylate pathway (the *pck* reporter) may indeed be indicative of the fluxes through those pathways.

### Switching to overflow metabolism and bimodal expression of the *acs* reporter

**Figure 5:**
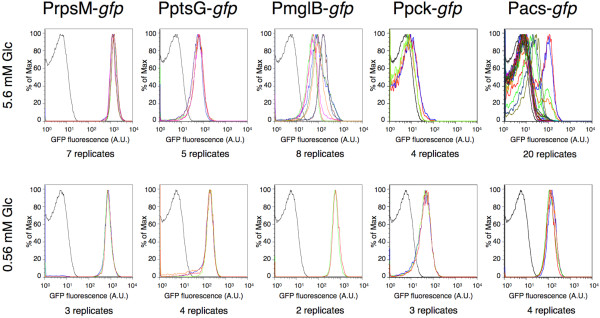
**Overflow metabolism in chemostat cultures at the intermediate growth rate D = 0.3 h**^**-1**^**.** Overflow metabolism occurs in chemostats with high concentration of glucose feed (5.6 mM Glc in the media). The distributions of fluorescence measurements corresponding to PrpsM-*gfp*, PptsG-*gfp*, PmglB-*gfp*, Ppck-*gfp* and Pacs-*gfp* are depicted in different colors presenting different replicates. The background fluorescence is plotted in black. High variability in the measurements of PmglB-*gfp* and Pacs-*gfp* (upper row) suggests that the chemostats were operated at the threshold of switching to overflow metabolism. Some replicates showed a bimodal distribution of the expression of the *acs* reporter in chemostats with 5.6 mM Glc in the feed. This suggests the presence of two phenotypic subpopulations with different expression patterns of *acs*: a first population down-regulates the *acs* expression (and possibly excretes acetate) and a second population expresses *acs* (and possibly takes up and utilizes acetate).

It has been shown that the excretion of acetate (overflow metabolism) occurs in chemostat populations at a dilution rate of about 0.3 h^-1^[22,44]. Increasing the concentration of glucose in the chemostat feed results in intensified production of acetate [39]. Our results support the existence of overflow metabolism at D = 0.3 h^-1^ in chemostats with high concentrations (5.6 mM) of glucose in the feed. Under these conditions, decreased expression of *acs* and *pck* reporters indicated that assimilation of acetate was reduced and gluconeogenesis was shut down (Figure 5). However, not all replicate cultures showed consistent patterns in the expression of transcriptional reporters. The expression of the reporters for *mglB* and *acs* was not consistent between different experiments, in contrast to the measurements for *rpsM*, *ptsG* and *pck* (Figure 5). This suggests that not all replicate cultures switched to the overflow metabolism, possibly due to the fact that the mini-chemostats were operated at the threshold of the expected switch to overflow metabolism.

Several replicates showed bimodal patterns of the expression of Pacs-*gfp* in well-mixed chemostat cultures. This is consistent with the idea that within clonal populations two phenotypically different subpopulations existed (Figure 5) – a first group of cells that presumably scavenged acetate and expressed the *acs* reporter and a second group that excreted acetate and thus down-regulated expression of *acs*. According to this scenario, the first subpopulation performed metabolic reactions indicative of carbon source limitation whereas the expression profiles of metabolic genes in the second subpopulation did not reflect glucose-limited conditions. These results potentially support the existence of phenotypic subpopulations that engage in acetate cross-feeding, as hypothesized above. However, it is also possible that both phenotypic subpopulations utilize glucose as the primary carbon source (since the expression of the *pck* reporter was only slightly above background, Figure 5) and the first subpopulation additionally recovers cytoplasmic acetate to increase intracellular levels of acetyl-AMP and acetylphosphate [44]. Future experiments with advanced continuous cultivation methods (e.g. accelerostat cultivation as described in [44]) would be valuable for further refining the environmental conditions where these two metabolic strategies co-exist.

## Conclusions

Many studies refer to glucose-limited chemostats as “simple conditions”, e.g. [28,29,48]. Even though glucose serves as a sole carbon source in these experiments, the metabolic regimes of the populations of *E. coli* are far away from “simple” [49]. Each cell within the bacterial population can take up glucose via five different transporters and metabolize it according to its needs for biomass building blocks and energy. Glucose is broken down to metabolic intermediates including acetate; acetate can be recovered in the central metabolic pathway, or it can be excreted and potentially then scavenged by other cells. Our results show that single cells within clonal population differ in their gene expression patterns and thus potentially in metabolic phenotypes when only glucose is supplied in the feed. This variation can arise through 1) different expression of glucose transporters (PtsG/Crr, MglBAC, etc.) between individual cells, 2) differences in utilization of acetate recovered within the cells and potentially, 3) uptake of excreted acetate. While the fluorescent reporter systems that we used in this study can provide insights into cell-to-cell variation in gene expression, and thus be used for the generation of hypotheses about metabolic variation, addition experiments are needed to test these hypotheses and to get direct insights into metabolic activities of cells growing on glucose.

*E. coli* is commonly used for the production of recombinant proteins and other valuable products, and the corresponding cultures are usually grown at high growth rates. High consumption of glucose is often associated with the excretion of acetate that inhibits recombinant protein production [44,45]. The findings presented here can provide a better understanding of the strategies involved in metabolizing glucose (as the only carbon-source component of the medium) and acetate that is subsequently produced during glucose utilization, and thus contribute to the development of new strategies for improving growth of industrial strains.

## Methods

### Bacterial strains

All *E.coli* K-12 MG1655 [50] strains with reporter plasmids used in this study are listed in Table 4. The strain containing the plasmid with the reporter Pacs-*gfp* was constructed as follows. A 858 bp-long intergenic region (comprising the region between *acs* and *nrfA* and the parts of the open reading frames) was amplified from the MG1655 chromosome using the primers Fwd_Pacs_XhoI 5’-*CCGCTCGAG*TAAGCTGAAGATACGGCGTGC-3’ and Rev_Pacs_BamHI 5’-*CGGGATCC*CCATCGGCATATAAATCGCCACC-3’ (italic parts of sequences are the restriction sites). The construct was cloned via XhoI/BamHI restriction into the plasmid containing the PptsG-*gfp* reporter [30] (thus swapping the existing *ptsG* promoter) and transformed into MG1655.

**Table 4:** **List of** ***E. coli*** **strains and plasmids**

**Strain name**	**Characteristics**	**Source**
MG1655	Wild-type *E.coli* K-12 F-, *λ*-, *ilvG*-, *rfb*-50, *rph*-1	Lab collection, [50]
DH5α	Strain for plasmid propagation F-, *glnV44*(AS), *λ*-, *deoR481*, *rfbC1*?, *gyrA96*(NalR), *recA1*, *endA1*, *thiE1*, *hsdR17*	Lab collection
MG1655 PptsG-*gfp*	*ptsG* reporter	Plasmid library [30]
MG1655 PmglB-*gfp*	*mglB* reporter	Plasmid library [30]
MG1655 PrpsM-*gfp*	*rpsM* reporter	Plasmid library [30]
MG1655 Ppck-*gfp*	*pck* reporter	Plasmid library [30]
MG1655 pUA66	Promoterless plasmid in MG1655	Plasmid library [30]
MG1655 Pacs-*gfp*	*acs* reporter	This study

### Growth media

The growth conditions are listed in Table 5. Briefly, *E.coli* strains were grown in minimal media supplemented with carbon source(s) in mini-chemostats [33] or in batch cultures at 37 °C.

**Table 5:** Growth conditions

**Experiment**	**Batch or chemostat**	**Supplemented carbon source**
Glucose environments	Chemostat, D = 0.15 h^-1^	0.56 mM Glc
	Batch	0.56 mM Glc
	Chemostat, D = 0.3 h^-1^	0.56 mM Glc
	Chemostat, D = 0.15 h^-1^	5.6 mM Glc
	Batch	5.6 mM Glc
Acetate environments	Chemostat, D = 0.15 h^-1^	0.56 mM Ac
	Batch	0.56 mM Ac
	Chemostat, D = 0.15 h^-1^	5.6 mM Ac
	Batch	5.6 mM Ac
Mixed-substrate environments	Chemostat, D = 0.15 h^-1^	2.8 mM Glc, 2.8 mM Ac
	Batch	2.8 mM Glc, 2.8 mM Ac
	Chemostat, D = 0.15 h^-1^	0.28 mM Glc, 0.28 mM Ac
	Batch	0.28 mM Glc, 0.28 mM Ac
*acs* expression	Chemostat, D = 0.15 h^-1^	11.2 mM Glc
	Chemostat, D = 0.15 h^-1^	2.8 mM Glc
Overflow metabolism	Chemostat, D = 0.3 h^-1^	5.6 mM Glc

Glc = glucose, Ac = acetate.

The cultures were grown in M9 minimal medium (Sigma-Aldrich) containing 47.76 mM Na_2_HPO_4_, 23.6 mM KH_2_PO_4_, 8.56 mM NaCl and 20.2 mM NH_4_Cl. 1 mL of 1 M MgSO_4_ (Fluka) and 100 μL of 1 M CaCl_2_ (Sigma-Aldrich) were added to 1 L of minimal medium. D(+)-glucose (Sigma) and/or sodium acetate (Fluka) were used as carbon source(s) and added to the desired concentration. The concentration of kanamycin sulfate (Sigma) was 50 μg/mL.

### Cultivation in the chemostats

Frozen clones were first streaked on LB agar (Sigma-Aldrich) plates to obtain single colonies. The agar plates contained 50 μg/mL of kanamycin for reporter strains [30]. A single colony was inoculated overnight in defined minimal medium (total 4 mL). 1 mL of these precultures was used to inoculate each mini-chemostat (total 5.5 mL) [33]. The minimal speed of the inflow pump corresponding to a dilution rate of D = 0.14 h^-1^ was increased in 2 or 3 steps until a dilution rate of D = 0.15 h^-1^ was reached after 24 h (using the peristaltic pump IPC-N from Ismatec, IDEX Health & Science, Germany). The airflow was maintained with the outflow pump (model IP from Ismatec, IDEX Health & Science, Germany) at 20 mL per minute with filter-sterilized water-saturated air [33]. Continuous formation of air-bubbles as well as small magnetic stirrer bars within the mini-chemostats ensured sufficient mixing of the bacterial cultures. The chemostats were harvested after 5 volume changes (one volume change every 6.67 hours) at the final dilution rate, i.e. after reaching the steady state [33] (Additional file 6: Figure S4).

For the experiments performed at D = 0.3 h^-1^ the total run-time was adjusted to the same number of volume changes as obtained with the experiments performed at D = 0.15 h^-1^.

### Batch cultivation

Frozen clones were first streaked on LB agar plates (containing kanamycin when needed). A single colony was inoculated overnight in defined minimal medium (total 4 mL). The overnight cultures were diluted 200-fold into 4 mL of minimal medium and grown for 2 hours before measured in the flow cytometer.

### Flow cytometry

We analyzed GFP fluorescence as a proxy for gene expression. For the strains grown in mini-chemostats, the GFP fluorescence was measured after 5 volume changes, which are required to reach steady state [33] (Additional file 6: Figure S4) but short enough to minimize the probability of mutations in the promoter region. GFP fluorescence was measured in the early exponential phase for the samples grown in the batch cultures.

All measurements were performed 2–5 times, as independent replicates coming from different overnight cultures. (For analysis of overflow metabolism we measured up to 20 replicates.)

We used the PAS-III flow cytometer (Partec, Muenster, Germany) equipped with 488 nm excitation laser. The green fluorescence was measured at 520 nm (FL1 channel), with the following settings – FSC (forward scatter): 270 V, SSC (side scatter): 210 V, FL1: 600 V, speed = 3, trigger on SSC.

### Data filtering

For each strain and all growth conditions, raw data were processed using FlowJo software version 8.8.7 (Tree Star, Inc.), and gated on 10,000-12,000 cells by using the autogating tool in the densest area of the pseudo-color plots of SSC vs. FSC. These gated cells were then used for the subsequent analysis. For analysis of the negative controls (strains with the promoterless plasmid pUA66 or wild-type MG1655) no gating was applied.

The cells were considered not to express a reporter when their fluorescence values were below the background fluorescence. The background fluorescence was defined as the mean value of the 99^th^ percentile of fluorescence intensities (Additional file 1: File S1) of the strain with the promoterless plasmid pUA66 (no gating applied) measured in various environments.

The fluorescence values for the cells within the gated populations were log_10_ transformed for the analysis, and thus we computed mean log expression and CV (coefficient of variation, the ratio between standard deviation and mean) of log expression.

### Influence of data filtering on the results

We restricted our analysis to the fraction of cells that were in similar physiological activity and size [31,51,52]. The cells were gated within a narrow range of defined flow cytometry parameters. We analyzed how the number of cells in the gated fraction influences the computation of mean and CV. One sample (the measurement of the strain harboring PmglB-*gfp* in the chemostats cultures at D = 0.15 h^-1^, with 5.6 mM Glc feed) was, therefore, gated 24 times (Additional file 7: Figure S5) while varying cell number in the range 5,000-20,000 cells.

### 2-NBDG assay

*E.coli* K-12 MG1655 [50] and the PptsG-*gfp* strain from the plasmid library [30] were used for these experiments. The strains were grown in the mini-chemostats [33] with minimal media supplemented with a sole carbon source (0.56 mM sodium acetate, 0.56 mM L-arabinose (Sigma-Aldrich), 0.56 mM D-glucose or 5.6 mM D-glucose). After 5 volume changes at D = 0.15 h^-1^, cells were harvested. Fluorescence was measured with the flow cytometer, as described above. PptsG-*gfp* fluorescence was measured immediately upon harvesting. MG1655 samples were incubated with 10 μM 2-NBDG (Molecular Probes, Life Technologies) for 5 minutes according to [34], and their fluorescence was measured directly afterwards.

### Ion chromatography

We analyzed glucose concentration by ion chromatography using Dionex DX-500 system with CarboPack PA10 carbohydrate column. The eluent was 200 mM NaOH, and the calibration curves were obtained by measuring glucose solutions of known concentration.

### Data analysis

The data were analyzed in SPSS statistical software version 19 and Microsoft Excel version 14.3.

## Abbreviations

Glc: Glucose; Ac: Acetate; D: Dilution rate; gfp: Green fluorescent protein; CV: Coefficient of variation; PTS: Phosphotransferase system; ABC: ATP-binding cassette; MFS: Major facilitator superfamily; PEP: Phosphoenolpyruvate; 2-NBDG: 2-[N-(7-nitrobenz-2-oxa-1,3-diazol-4-yl)amino]-2-deoxy-D-glucose; FSC: Forward scatter; SSC: Side scatter; A.U: Arbitrary unit.

## Competing interests

The authors declare that they have no competing interests.

## Authors’ contributions

Conceived and designed the experiments: NN MA. Performed the experiments: NN TB. Analyzed the data: NN TB MA. Wrote the manuscript: NN MA. All authors read and approved the final manuscript.

## Supplementary Material

Additional file 1: File S1 Flow cytometry data. Click here for file

Additional file 2: Figure S1 Variation in the expression of *ptsG*, *mglB* and *rpsM* reporters across different environments. The CV of log expression of PptsG-*gfp* (green), PmglB-*gfp* (blue) and PrpsM-*gfp* (red) was plotted against the mean log expression. Power regression was fitted to each dataset corresponding to the expression of the same reporter across different environments. The individual curves of variation in the expression of *ptsG* and *rpsM* reporters showed negative associations between the mean expression and the variation of expression across environments, whereas the *mglB* reporter showed a positive association. Click here for file

Additional file 3: Text S1 Analysis of expression of fluorescent reporters in glucose-acetate mixtures. Click here for file

Additional file 4: Figure S2 Reporter expression in mixed-substrate environments. Expression of *ptsG*, *mglB* and *acs* reporters was measured in chemostats (D = 0.15 h^-1^) in mixed-substrate environments supplemented with 0.28 mM Glc and 0.28 mM Ac (green), or 2.8 mM Glc and 2.8 mM Ac (blue). The distributions were plotted together with the measurements of the reporter expression in the environments with only glucose in the feed (0.56 mM Glc – orange, and 5.6 mM Glc – red). The fluorescence of the promoterless strain is presented in black. Click here for file

Additional file 5: Figure S3 Expression of the *pck* reporter in different chemostat and batch conditions. Ppck-*gfp* fluorescence (indication of flux to gluconeogenesis) was measured in bacterial populations grown in chemostats (D = 0.15 h^-1^) and batch environments supplied with minimal media supplemented with only D-glucose, only sodium acetate or D-glucose plus sodium acetate. Again, background fluorescence is the fluorescence of the promoterless strain, depicted in black. The expression of the *pck* reporter was decreased in the exponential phase in glucose batch cultures in comparison to carbon-limited chemostats. Click here for file

Additional file 6: Figure S4 Changes in *gfp* expression prior of reaching theoretical steady-state. Pacs-*gfp* fluorescence was measured for five independent replicates growing on different concentration of glucose in the feed. At time point of 0 hours, chemostat experiments were started at a minimal dilution rate of D = 0.14 h^-1^. After 24 hours, dilution rates were increased to D = 0.15 h^-1^. The fluorescence plots show *gfp* distribution in bacterial populations without gating, together with fluorescence of the promoterless strain depicted in black. All independent replicates showed reproducible measurements of GFP fluorescence after 3.6 volume turnovers at D = 0.15 h^-1^. Click here for file

Additional file 7: Figure S5 Influence of size of the gate on the mean and CV. The strain carrying PmglB-*gfp* was grown in chemostats (at D = 0.15 h^-1^, with 5.6 mM Glc) and analyzed with flow cytometry. A) For subsequent analysis, the cells were gated using the autogating tool (FlowJo, Tree Star, Inc.) in the densest area of the pseudo-color plots of SSC vs. FSC. B) The gating was performed 24 times to capture between 5,000-20,000 cells, and the resulting distributions of GFP fluorescence were plotted. This yielded mean log expression of 2.69 ± 0.005 (mean ± standard deviation) and CV was 0.13 ± 0.0014. This suggests that the results for mean expression and CV deviated less than 1% when gate size was varying 4-fold. Our gate size varied maximally 1.2-fold when analyzing 10,000-12,000 cells, therefore the slight differences in the gate size should minimally influence the computation of mean and CV. Click here for file
