# Supplementary figures and images for: Analysis of fluorescent reporters indicates heterogeneity in glucose uptake and utilization in clonal bacterial populations
Source: BMC Microbiol. 2013 Nov 15;13:258. doi: 10.1186/1471-2180-13-258 (PMC3840653; doi:10.1186/1471-2180-13-258)

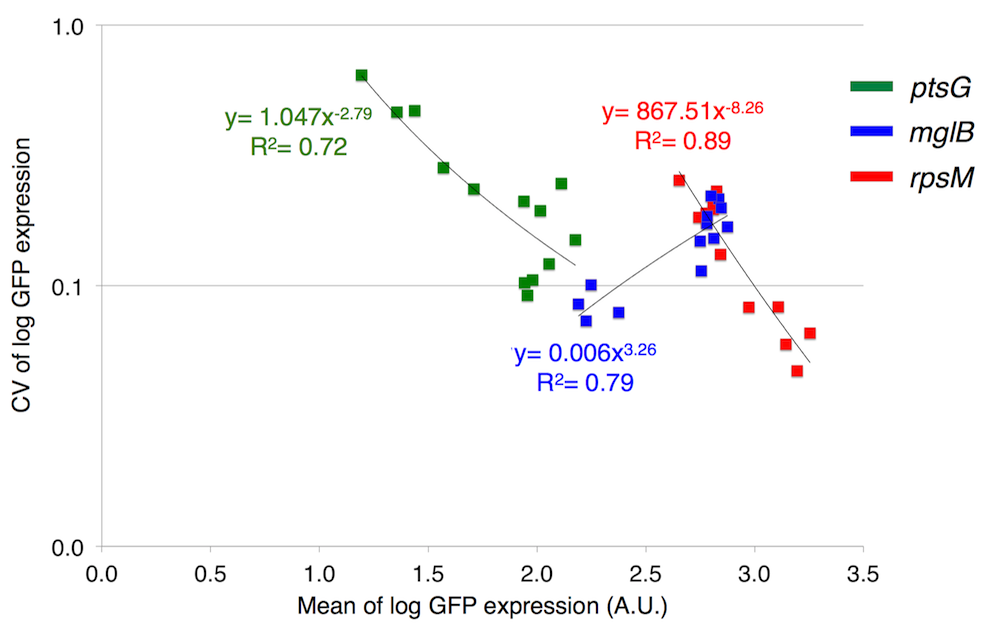

Supplement: Additional file 2: Figure S1 — Variation in the expression of ptsG, mglB and rpsM reporters across different environments. The CV of log expression of PptsG-gfp (green), PmglB-gfp (blue) and PrpsM-gfp (red) was plotted against the mean log expression. Power regression was fitted to each dataset corresponding to the expression of the same reporter across different environments. The individual curves of variation in the expression of ptsG and rpsM reporters showed negative associations between the mean expression and the variation of expression across environments, whereas the mglB reporter showed a positive association. [file 1471-2180-13-258-S2.tiff]

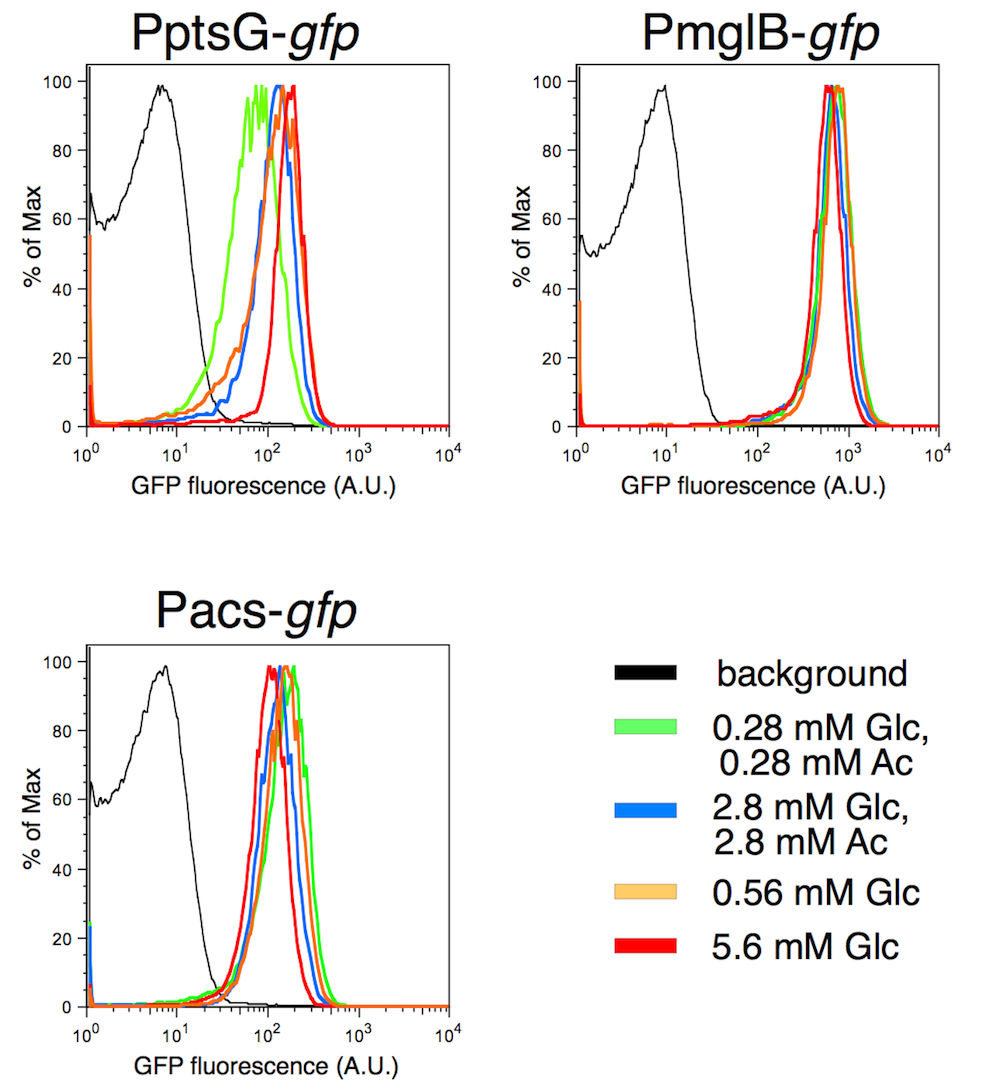

Supplement: Additional file 4: Figure S2 — Reporter expression in mixed-substrate environments. Expression of ptsG, mglB and acs reporters was measured in chemostats (D = 0.15 h-1) in mixed-substrate environments supplemented with 0.28 mM Glc and 0.28 mM Ac (green), or 2.8 mM Glc and 2.8 mM Ac (blue). The distributions were plotted together with the measurements of the reporter expression in the environments with only glucose in the feed (0.56 mM Glc – orange, and 5.6 mM Glc – red). The fluorescence of the promoterless strain is presented in black. [file 1471-2180-13-258-S4.tiff]

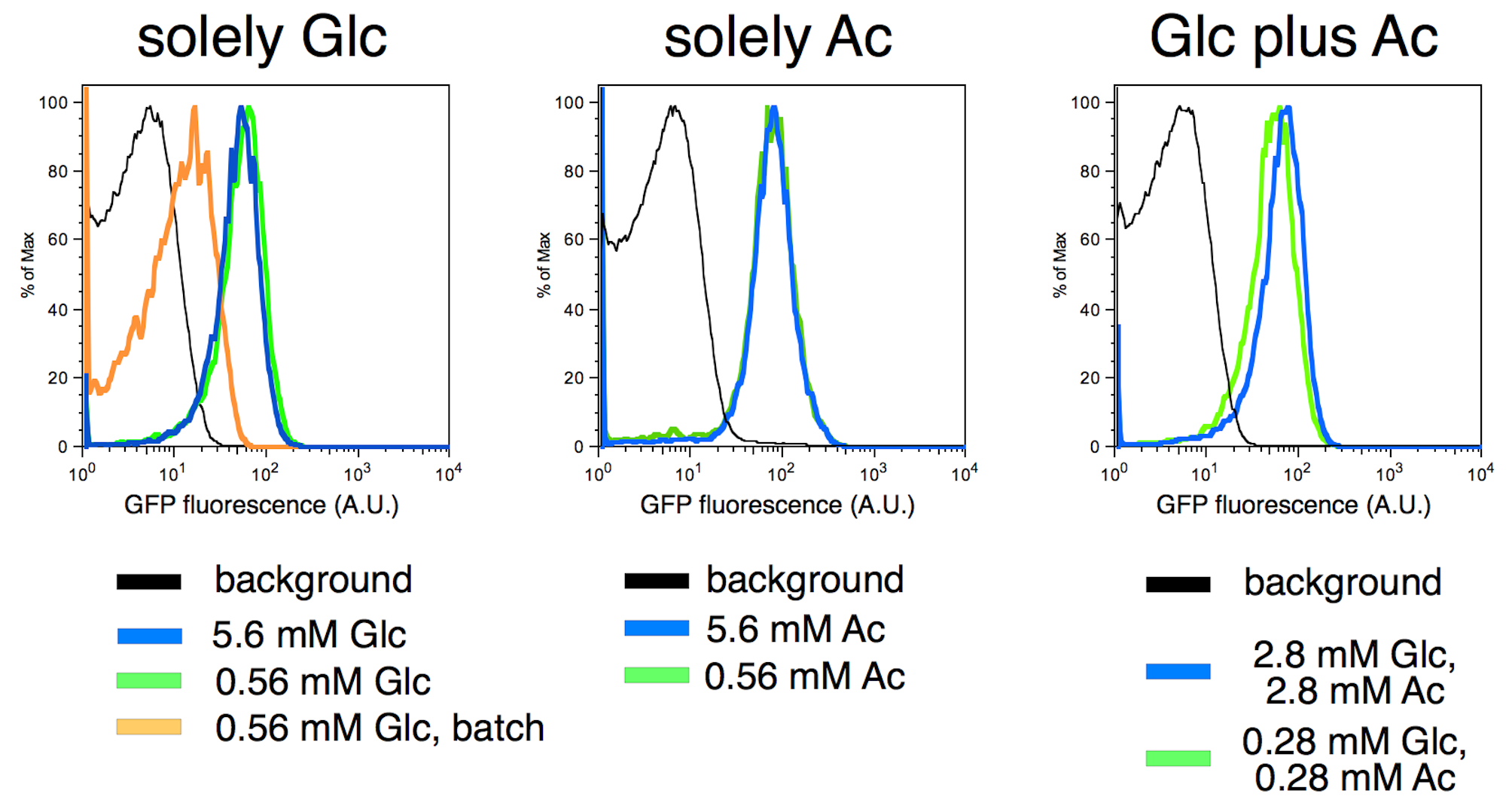

Supplement: Additional file 5: Figure S3 — Expression of the pck reporter in different chemostat and batch conditions. Ppck-gfp fluorescence (indication of flux to gluconeogenesis) was measured in bacterial populations grown in chemostats (D = 0.15 h-1) and batch environments supplied with minimal media supplemented with only D-glucose, only sodium acetate or D-glucose plus sodium acetate. Again, background fluorescence is the fluorescence of the promoterless strain, depicted in black. The expression of the pck reporter was decreased in the exponential phase in glucose batch cultures in comparison to carbon-limited chemostats. [file 1471-2180-13-258-S5.tiff]

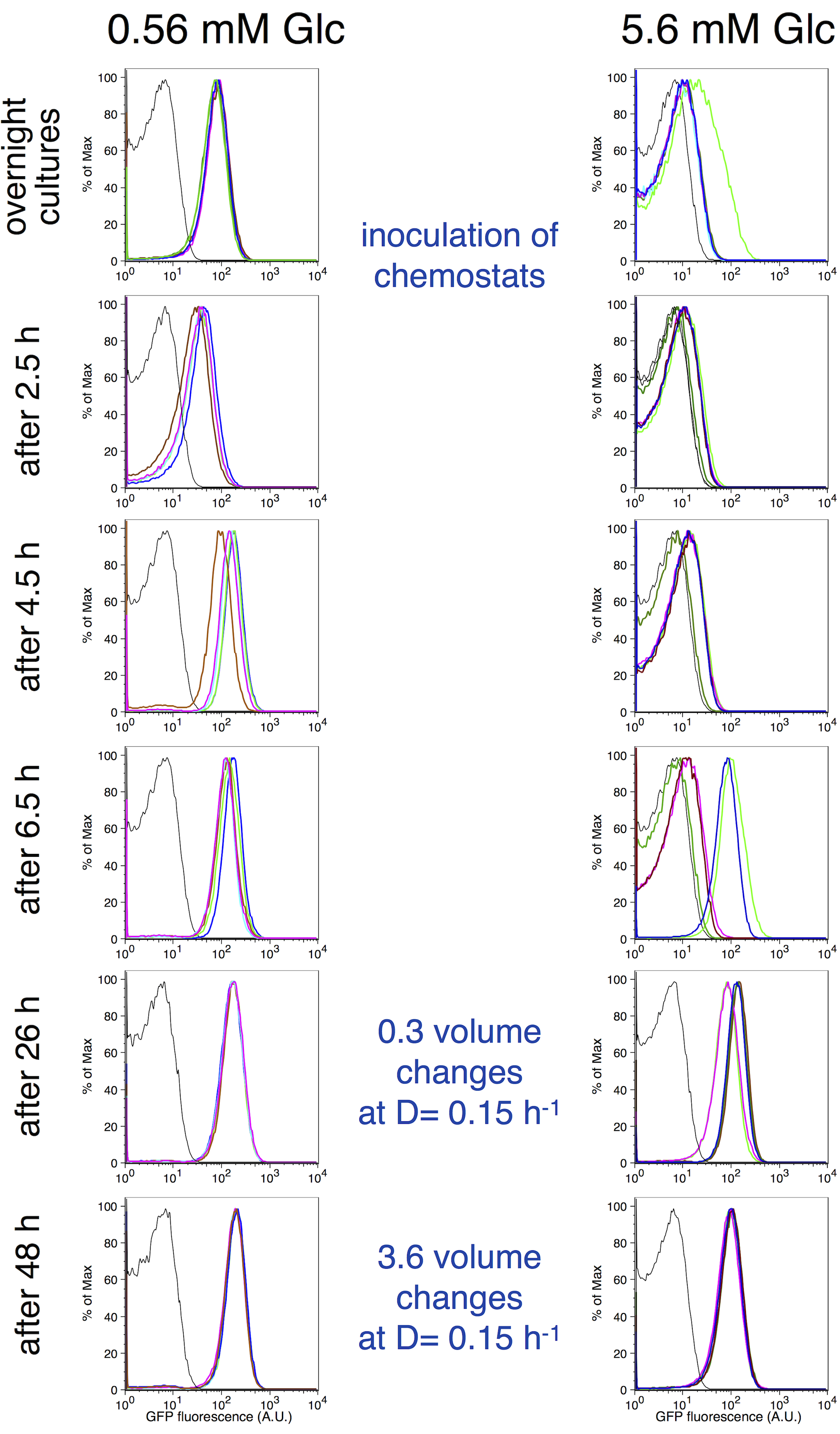

Supplement: Additional file 6: Figure S4 — Changes in gfp expression prior of reaching theoretical steady-state. Pacs-gfp fluorescence was measured for five independent replicates growing on different concentration of glucose in the feed. At time point of 0 hours, chemostat experiments were started at a minimal dilution rate of D = 0.14 h-1. After 24 hours, dilution rates were increased to D = 0.15 h-1. The fluorescence plots show gfp distribution in bacterial populations without gating, together with fluorescence of the promoterless strain depicted in black. All independent replicates showed reproducible measurements of GFP fluorescence after 3.6 volume turnovers at D = 0.15 h-1. [file 1471-2180-13-258-S6.tiff]

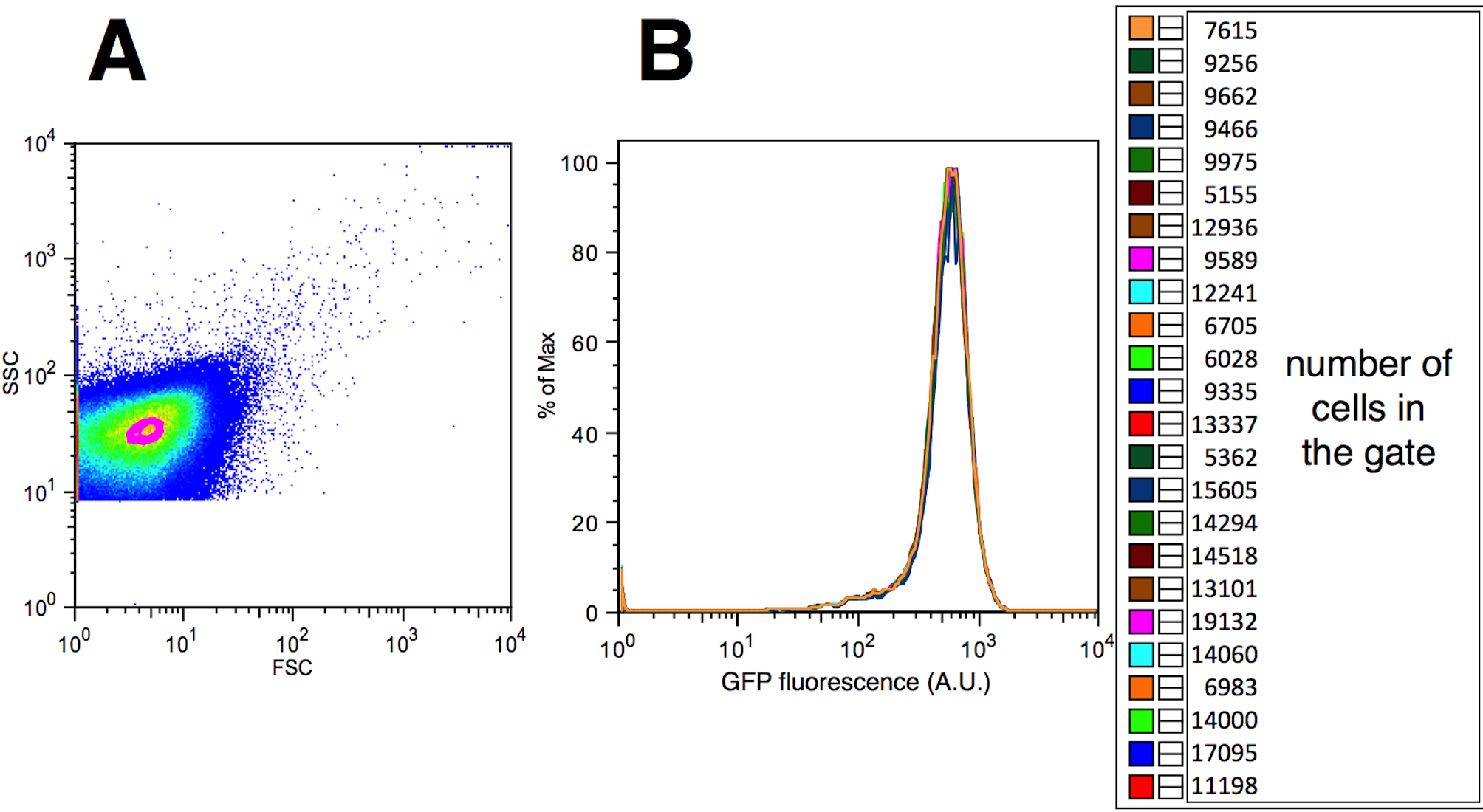

Supplement: Additional file 7: Figure S5 — Influence of size of the gate on the mean and CV. The strain carrying PmglB-gfp was grown in chemostats (at D = 0.15 h-1, with 5.6 mM Glc) and analyzed with flow cytometry. A) For subsequent analysis, the cells were gated using the autogating tool (FlowJo, Tree Star, Inc.) in the densest area of the pseudo-color plots of SSC vs. FSC. B) The gating was performed 24 times to capture between 5,000-20,000 cells, and the resulting distributions of GFP fluorescence were plotted. This yielded mean log expression of 2.69 ± 0.005 (mean ± standard deviation) and CV was 0.13 ± 0.0014. This suggests that the results for mean expression and CV deviated less than 1% when gate size was varying 4-fold. Our gate size varied maximally 1.2-fold when analyzing 10,000-12,000 cells, therefore the slight differences in the gate size should minimally influence the computation of mean and CV. [file 1471-2180-13-258-S7.tiff]
