# Supplementary material for: Analysis of fluorescent reporters indicates heterogeneity in glucose uptake and utilization in clonal bacterial populations
Source: BMC Microbiol. 2013 Nov 15;13:258. doi: 10.1186/1471-2180-13-258 (PMC3840653; doi:10.1186/1471-2180-13-258)
Supplement: Additional file 3: Text S1 — Analysis of expression of fluorescent reporters in glucose-acetate mixtures. [file 1471-2180-13-258-S3.pdf]

Additional File 3 – Text S1

Nikolic et al. “Analysis of fluorescent reporters indicates heterogeneity in glucose uptake and utilization in clonal bacterial populations”

Analysis of expression of fluorescent reporters in glucose-acetate mixtures

We were also interested in heterogeneity in the expression of the genes in environments with glucose and acetate in the feed. Our goal was to assess whether heterogeneity in mixed-substrate environments is higher than in glucose-feed conditions. We measured the expression of the reporters for glucose transporter systems PtsG and MglBAC in medium that was initially supplemented with both glucose and acetate. The expression of the *ptsG* reporter was decreased in conditions with 2.8 mM Glc and 2.8 mM Ac in the feed solution, in comparison to the expression in medium supplemented only with glucose, 5.6 mM Glc feed (Table 1, Additional File 1 – File S1, Additional File 4 – Figure S2). In contrast, Pacs-*gfp* showed higher expression level when the strain was grown the environment containing glucose and acetate in the feed solution. The expression of PmglB-*gfp* only exhibited minor changes when grown in single-glucose or mixed-substrate environments under these chemostat conditions, at D= 0.15 h^{-1} (Table 1).

When comparing variation in the expression between reporters, in general all reporters for the metabolic genes (*ptsG*, *mglB*, *acs*) and the ribosomal gene (*rpsM*) showed higher variation when bacteria were grown in glucose-acetate environments compared to glucose-feed conditions (Table 2, Table 3). As the expression of the ribosomal reporter follows the same pattern, we speculate that increased variation of all tested reporters is not a direct consequence of altered activities of metabolic promoters in single cells but a result of altered general physiological state of the population.

Since down-regulation of *ptsG* and up-regulation of *acs* are indicators of growth on acetate [40], we assume that under mixed-substrate conditions in the chemostats at D= 0.15 h^{-1}, both glucose and acetate might be simultaneously utilized. It remains an open question what are actual metabolic phenotypes under such conditions: whether potentially consumed acetate comes directly from the supplied medium, whether it is recovered from cellular metabolism, or whether the cells scavenge exogenous acetate excreted by other cells.
